# Elamipretide (SS-31) Improves Functional Connectivity in Hippocampus and Other Related Regions Following Prolonged Neuroinflammation Induced by Lipopolysaccharide in Aged Rats

**DOI:** 10.3389/fnagi.2021.600484

**Published:** 2021-03-01

**Authors:** Yang Liu, Huiqun Fu, Yan Wu, Binbin Nie, Fangyan Liu, Tianlong Wang, Wei Xiao, Shuyi Yang, Minhui Kan, Long Fan

**Affiliations:** ^1^Department of Anesthesiology, Xuanwu Hospital, Capital Medical University, Beijing, China; ^2^Department of Anatomy, Capital Medical University, Beijing, China; ^3^Institue of High Energy Physics, Chinese Academy of Sciences, Beijing, China

**Keywords:** lipopolysaccharide, neuroinflammation, functional connectivity, elamipretide, cognition

## Abstract

Neuroinflammation has been recognized as a major cause for neurocognitive diseases. Although the hippocampus has been considered an important region for cognitive dysfunction, the influence of hippocampal neuroinflammation on brain functional connectivity (FC) has been rarely studied. In this study, lipopolysaccharide (LPS) was used to induce systemic inflammation and neuroinflammation in the aged rat brain, while elamipretide (SS-31) was used for treatment. Systemic and hippocampal inflammation were determined using ELISA, while astrocyte responses during hippocampal neuroinflammation were determined by interleukin 1 beta (IL-1β)/tumor necrosis factor alpha (TNFα) double staining immunofluorescence. Oxidative stress was determined by reactive oxidative species (ROS), electron transport chain (ETC) complex, and superoxide dismutase (SOD). Short- (<7 days) and long-term (>30 days) learning and spatial working memory were tested by the Morris water maze (MWM). Resting-state functional magnetic resonance imaging (rs-fMRI) was used to analyze the brain FC by placing seed voxels on the left and right hippocampus. Compared with the vehicle group, rats with the LPS exposure showed an impaired MWM performance, higher oxidative stress, higher levels of inflammatory cytokines, and astrocyte activation in the hippocampus. The neuroimaging examination showed decreased FC on the right orbital cortex, right olfactory bulb, and left hippocampus on day 3, 7, and 31, respectively, after treatment. In contrast, rats with SS-31 treatment showed lower levels of inflammatory cytokines, less astrocyte activation in the hippocampus, and improved MWM performance. Neuroimaging examination showed increased FC on the left-parietal association cortex (L-PAC), left sensory cortex, and left motor cortex on day 7 with the right flocculonodular lobe on day 31 as compared with those without SS-31 treatment. Our study demonstrated that inhibiting neuroinflammation in the hippocampus not only reduces inflammatory responses in the hippocampus but also improves the brain FC in regions related to the hippocampus. Furthermore, early anti-inflammatory treatment with SS-31 has a long-lasting effect on reducing the impact of LPS-induced neuroinflammation.

## Introduction

Neuroinflammation has been proved to be a major cause for post-operative cognitive dysfunction (POCD) and other perioperative neurocognitive disorders (Subramaniyan and Terrando, 2019). As a crucial region to learning and memory, the hippocampus has traditionally been considered as a classic region for studying neuroinflammation and neurodegenerative diseases in many clinical studies and preclinical experiments. The recent studies demonstrated that although various regions may also participate, the hippocampus, both morphology and function, is one of the most sensitive regions that is affected by aging, neuroinflammation, and chronic stress (Fjell et al., 2014; Bartsch and Wulff, 2015). Meanwhile, clinical studies have reported that the disruption of the brain–blood barrier (BBB), an early event in the aging brain, begins in the hippocampus (Montagne et al., 2015). These findings suggest that the hippocampus in the aging brain is more likely to be affected by peripheral inflammatory cytokines triggered by surgery and other perioperative stress events. Therefore, the hippocampus is a brain region of great importance in understanding the development and molecular mechanisms of cognitive dysfunction.

Acute and chronic inflammation in the hippocampus has already been associated with cognitive dysfunction in neurocognitive diseases (Schwalm et al., 2014; Chesnokova et al., 2016). In our previous work, we reported the specific roles of interleukin-1beta (IL-1β) secretion and the nuclear factor-kappa B (NF-κB) signaling pathway in the long-term astrocyte activation after lipopolysaccharide (LPS) exposure in aged rats (Fu et al., 2014; Kan et al., 2016). These results suggest that astrocyte-derived inflammatory cytokines are important in the long-term neuroinflammation in aged rats, contributing to neuron homeostasis, BBB function, improving local synapse transmission and plasticity, and in the regulation of structural remodeling (Abbott et al., 2006; Santello et al., 2019). The aged brain is known to form many more neurotoxic A1 type astrocytes in response to LPS in neuroinflammation (Clarke et al., 2018); thus, the aged rats are more suitable to be used for studying mechanisms for neuroinflammation induced by POCD. However, a variety of preclinical, clinical, and neuroimaging studies has indicated that the hippocampus is not only the region affected by neurocognitive disease (Teipel et al., 2015; Mitchell et al., 2018; Zhang et al., 2019), besides the hippocampus neuroinflammation, the brain functions in other related regions may also be affected in disease development.

Oxidative stress has been reported to coexisted with neuroinflammation, and mitochondria play an important role in cognitive dysfunction (Wu et al., 2017; Zhao et al., 2019). Recent study showed that reducing the level of mitochondrial reactive oxygen species (mtROS) triggered by LPS also reduced the inflammatory responses in BV-2 cell (microglia cell line) (Park et al., 2015). Meanwhile, there are also studies supporting the evidence that mtROS also induce inflammatory responses in the astrocyte (Alfonso-Loeches et al., 2014). Elamipretide (SS-31), a mitochondria targeted antioxidant, functions by removing excessive reactive oxygen species (ROS) produced during the oxidative stress. However, whether reducing the ROS level also reduces inflammatory responses in the astrocytes and improves the long-term cognitive function in aged brains still need further investigation.

Resting state functional magnetic resonance imaging (rs-fMRI) enabled a non-invasive method for studying the brain function in each pathological phase for the disease development *in vivo* (Lopes et al., 2017; Hardcastle et al., 2019; Passamonti et al., 2019). Using a seed voxel, the hippocampus revealed decreased functional connectivity (FC) in Alzheimer's disease (AD) (Qian et al., 2019), mild cognitive impairment (MCI) (Chirles et al., 2017), and POCD (Browndyke et al., 2017), suggesting that apart from the hippocampus, other brain regions are also affected because they show decreased correlations with the hippocampus. Recently, rs-fMRI also has been conducted to detect the long-term change of the brain function in rats after repeated mild traumatic brain (Pham et al., 2021). However, the cellular and molecular mechanisms underlying these changes are unclear. In rodents, recent studies found that neuroinflammation, microglia, and astrocyte activation, and changes in spatial working memory coexisted with alteration in the brain function (Nasrallah et al., 2016; Griffin et al., 2019), indicating that rodent models are highly attractive options for simultaneous study of neuroimaging changes and molecular mechanisms.

In the present study, we investigated the effects of hippocampal neuroinflammation on the brain FC in aged rats by removing excessive oxidative stress after LPS exposure. We combined neuroimaging and neuroscience to assess both the hippocampal neuroinflammation and brain function in regions related to cognition. The model was chosen based on our previous findings that LPS exposure causes long-term neuroinflammation characterized by cognitive dysfunction and astrocyte activation in aged rats (Fu et al., 2014; Kan et al., 2016). Because elamipretide (SS-31), has been reported to be able to improve cognitive dysfunction and inflammatory responses in the hippocampus caused by LPS and inhalational anesthetics (Wu et al., 2017; Zhao et al., 2019), it was used to inhibit inflammatory response after LPS exposure. We hypothesize that anti-inflammatory protection provided by SS-31 treatment will both improve the short-term (within 7 days) and long-term (after 30 days) brain FC and ameliorate the hippocampal neuroinflammation after LPS exposure.

## Materials and Methods

### Ethical Approval

This experiment was approved by the ethical committee of Capital Medical University (Approved number: AEEI-2018-166) and complied with the Guide for the Care and Use of Laboratory Animals prepared by the Institute of Laboratory Animal Resources and published by the National Institute of Health. All efforts were used to minimize the pain and suffering of animals.

### Animals and Treatments

**Figure 1:**
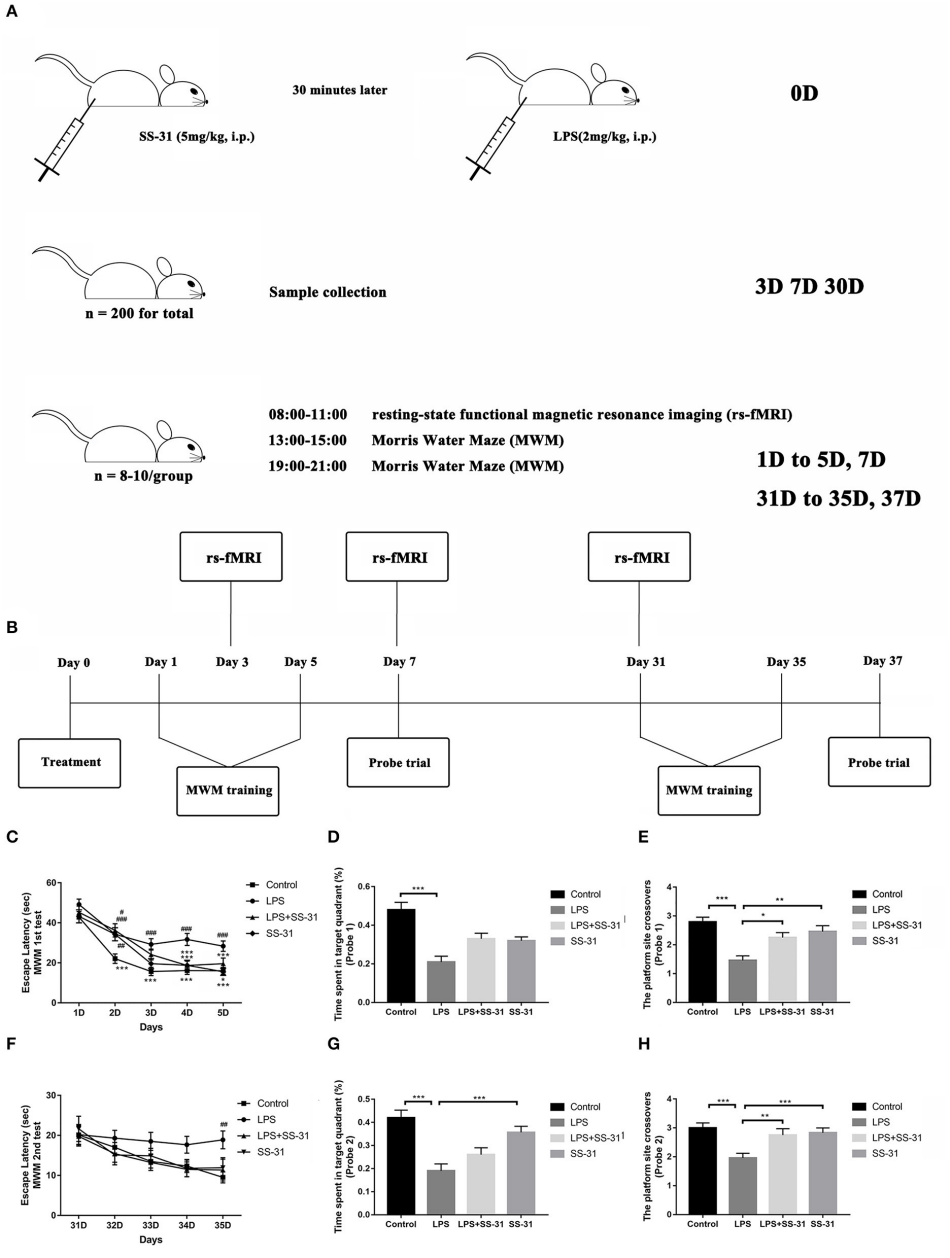
Elamipretide (SS-31) improved cognitive dysfunction induced by lipopolysaccharide (LPS) in the aged rat brain. **(A)** Detailed processing line. **(B)** Morris water maze (MWM) training protocol. Spatial acquisition trials and probe trials were performed on both short term (day 1–7) and long term (day 31–37). **(C)** Rats treated with LPS showed a significant prolonged escape latency compared with those in vehicle (V) group and SS-31 (S) group from day 2 to 5, which was improved by SS-31 treatment from day 4 in LPS + SS-31 (L + S) group. **(D,E)** Rats treated with LPS showed reduced time spent in the target quadrant and fewer crossovers to this quadrant compared with V group. The reduction in crossovers was improved by SS-31 treatment. **(F)** In the long term, a significant difference in escape latency was observed on day 35 with rats treated with LPS seemed to have prolonged escape latency. **(G,H)** Rats treated with LPS spent significantly less time in the target quadrant compared to rats in the V and S groups, as well as showing significantly fewer crossovers than all other groups. Data are presented as mean ± SEMs, and escape latency was analyzed by two-way ANOVA for repeated measurements with the Bonferroni *post-hoc* analysis. Time spent in the target quadrant and crossovers were analyzed by one-way ANOVA followed with the Bonferroni *post-hoc* analysis. ^#^*p* < 0.05, ^*##*^*p* < 0.01, ^*###*^*p* < 0.001 as indicated or vs. V; *p < 0.05, **p < 0.01, ***p < 0.001 vs. L group.

two-hundred and forty male rats (Wistar, 20 months old, 500–700 g, SPF grade, Chengdu Dossy Experimental Animals Co., Ltd., Sichuan Province, China) were used in this study. All rats were housed in standardized and controlled laboratory conditions (21–25°C in temperature and 50–60% in humidity on a 12-h light/dark cycle with food and water provided *ad libitum*). The detailed processing line is shown in Figure 1A.

Rats were randomly divided into four groups:

1. Vehicle group (V group, *n* = 50): Rats were treated with 0.9% normal saline intraperitoneal (i.p.) injection.
2. SS-31-treated group (S group, *n* = 50): Rats were treated with SS-31 (5 mg/kg i.p. at a volume of 0.4 ml/kg, dissolved in normal saline, and synthesized by China Peptides Co., Ltd, Shanghai, China).
3. LPS-treated group (L group, *n* = 70): Rats were treated with LPS (2 mg/kg i.p., dissolved in normal saline, 055:B5, Sigma-Aldrich, St. Louis, MO, USA).
4. LPS + SS-31-treated group (L + S group, *n* = 70): Rats were treated with SS-31 (5 mg/kg) for 30 min before the LPS injection (both i.p. injections).

The doses of LPS and SS-31 were determined based on the previous studies (Fu et al., 2014; Wu et al., 2016). All time points were selected according to our previous work (Fu et al., 2014; Kan et al., 2016).

### Tissue Preparation

A cohort of rats were randomly selected in each group and sacrificed at 3, 7, and 30 days (*n* = 5 per time point per group) after treatment. Under deep isoflurane anesthesia (Forene, Abbott Laboratories, Queenborough, UK), the rat brains were rapidly dissected following decapitation. All dissections were performed on a chilled-ice glass plate. The entire dissected brain was immediately mounted in optimum cutting temperature (OCT) compound (Sakura Finetek USA, Inc., Torrance, CA, USA), frozen in liquid nitrogen, and then stored at −80°C subsequent to immunofluorescence. Another cohort of rats (n = 5 per time point per group) were sacrificed at the same time points mentioned above for measuring inflammatory cytokines in hippocampus. The blood samples were extracted from the inferior vena cava. After clotting at 4°C, the blood sample was centrifuged for 20 min at 1,000 × *g*. The serum was collected and stored at −80°C for subsequent ELISA analysis.

For another cohort of rats (*n* = 5 per time point per group), the hippocampus was collected for doing real-time PCR (RT-PCR) and mitochondria isolating (the left for RT-PCR and the right for isolating mitochondria). The hippocampus mitochondria were isolated using the Mitochondria Isolation Reagent Kit (C3606, Beyotime Institute of Biotechnology Co., Shanghai, China) according to the manufacturer's instructions. All the procedures were done on chilled cold ice, and the whole procedure was finished within 1 h. After isolation, the mitochondria sediments were resuspended by appropriate amount of Mitochondrial Stock Solution (GMS 12198.2, GenMed Scientific, Shanghai, China).

### Immunofluorescence and Microscopy

The coronal hippocampus sections were cut at a thickness of 20 μm with a freezing microtome (CM1850, Leica Microsciences, Mannheim, Germany). The sections were selected according to the landmarks corresponding to the Paxino & Watson's rat brain atlas (bregma −2.80 to −3.60) for subsequent immunofluorescence staining. The selected sections were first fixed in ice-cold 4% paraformaldehyde (CM-0055, Lifeline Cell Technology, Frederick, MD, USA) for 15 min then washed with 10 mM phosphate buffered saline (PBS; ZLI-9062, Zhongshan Goldbridge Biotech, Co, Ltd, Beijing, China) for three times. After permeabilizing using 0.3% Triton-X 100 (T9284, Sigma-Aldrich, St Louis, MO, USA) for 1 h at room temperature, sections were blocked by 10% horse serum (8178102 Gibco New Zealand Origin) for 1 h. The sections were then incubated in primary antibodies for 2 h at room temperature and then overnight at 4°C: goat anti-IL-1β immunoglobulin G (IgG) (1:100, AF-501-NA, R&D Systems, Inc., Minneapolis, MN, USA), goat anti-tumor necrosis factor alpha (anti-TNFα) IgG (1:100, AF-510-NA, R&D Systems, Inc., Minneapolis, MN, USA), and mouse anti-glial fibrillary acidic protein (anti-GFAP)-IgG (1:1,000, MAB360, Millipore Corp., Billerica, MA, USA), respectively. The sections were washed with PBS for three times and then incubated in secondary antibodies in a dark room at room temperature for 2 h: Alexa Fluor 594 donkey anti-goat IgG (1:500, A11058, Invitrogen, Paisley, UK) and Alexa Fluor 488 donkey anti-mouse IgG (1:500, A21202, Invitrogen, Paisley, UK). Finally, all sections were counter-stained and then covered with fluorescent mounting medium with 4′,6-diamidino-2-phenylindole (DAPI; ZLI-9557, Zhongshan Goldbridge Biotech, Co., Ltd, Beijing, China) and anti-fading reagents. For negative control treatments, the primary and secondary antibodies were replaced by PBS, while other procedures remained the same.

Fluorescent images were analyzed using a confocal microscopy (Leica TCS2, Leica Microsystems, Mannheim, Germany) and image analyzing system (Optimas 6.5, CyberMetrics, Scottsdale, AZ, USA) by an independent observer who was blinded from the entire study. Cells were counted in the hippocampus DG region using the NIH ImageJ software (National Institutes of Health, Bethesda, MD, USA), from which the percentages of double stained GFAP (IL-1β and TNFα positive) in total GFAP were calculated.

### ELISA Measurement

Inflammatory factors, including IL-1β and TNFα in serum, were measured using ELISA kits (RLB00 and RTA00, R&D Systems, Inc., Minneapolis, MN, USA) following the manufacturer's instructions. The proteins collected from the hippocampus were first analyzed using the BCA Protein Assay Kit (23227, Thermo Fisher Scientific, Waltham, MA, USA).

### mRNA and RT-PCR

Total RNA was extracted from the hippocampus using Trizol (Invitrogen, Paisley, UK) and purified by RNase Away Reagent (catalog number 18270466, Invitrogen, Paisley, UK). The total amount of RNA was measured by NanoDrope 2000 (Thermo Fisher Scientific, Waltham, MA, USA), and the building of cDNA was synthesized using the M-MLV Reverse Transcriptase Kit (Promega, Madison, WI, USA) according to the manufacturer's instructions. The cDNA amplification was carried out using ABI 7500 Fast Real-Time PCR System (Applied Biosystems, Waltham, MA, USA) for 45 cycles (each for a duration of 2 min at 94°C, annealing for 5 s at 94°C and for 30 s at 60°C and finally extension for 10 min at 72°C). The primer sequences and amplification for IL-1β and TNFα were described in Table 1. The level of mRNA expression was evaluated by the SYBR Green detection method. Data were analyzed using the 2-ΔΔCt method by the expression of target gene in the rat hippocampus that is corrected by the expression of endogenous control (β-actin) and relative to the vehicle group.

**Table 1:** Sequences of primers in RT-PCT.

**mRNA**	**Forward primer (5^′^ → 3^′^)**	**Reverse primer (5^′^ → 3^′^)**
IL-1β	5′-ATGAGAGCATCCAGCTTCAAATC-3′	5′-CACACTAGCAGGTCGTCATCATC-3′
TNFα	5′-CAAGAGCCCTTGCCCTAA-3′	5′-CAGAGCAATGACTCCAAAGTA-3′
β-actin	5′-CCCATCTATGAGGGTTACGC-3′	5′-TTTAATGTCACGCACGATTTC-3′

### Oxidative Stress

The mtROS were measured using the ROS Assay Kit (GMS 10104.1, GenMed Scientifics, Shanghai, China) according to the manufacturer's instructions. The serum and hippocampus SOD activity were measured using the SOD Assay Kit (Jiacheng Biological Technology Co., Ltd., Nanjing, China) according to the manufacturer's instructions. The enzyme activity was converted to units per milligram of protein. One unit of SOD activity was defined as the amount of that reduced the absorbance at 450 nm by 50%. The levels of electron transport chain (ETC) components—I/III/IV were also measured using the ETC Analyzing Kit (GMS 50007 for ETC-I, GMS 50009 for ETC-III and GMS 50010 for ETC-IV, GenMed Scientifics, Shanghai, China). All the above procedures were conducted on ice in a dark room. The results were collected by a microplate reader (SpectraMax iD5, Molecular Devices, San Jose, CA, USA).

### Resting-State fMRI Acquisition

On day 3, 7, and 31 after treatment, 8–10 rats in each group were randomly selected for rs-fMRI data acquisition. The animal MRI measurements were performed using the 7.0-T Bruker PharmaScan System (70/16 PharmaScan, Bruker BioSpin GmbH, Rheinstetten, Germany), operated *via* the ParaVision 5.1 software. The same coils, including a rat brain surface coil and a quadrature resonator volume coil, were adopted in all the rats. Shimming was optimized for the cerebrum (40 × 40 × 40 mm voxel) using a three-dimensional field map-based automatic shimming method.

The animal anesthesia was conducted as Lu et al. (2012) described elsewhere. Briefly, animals were first anesthetized using isoflurane (3%) in oxygen and air (1:1) followed by dexmedetomidine (0.015 mg/kg, intramuscular injection) in an anesthesia induction chamber. When the animal was fully anesthetized, it was gently fixed in prone position on the MRI scanning bed, and anesthesia was maintained using isoflurane with enriched oxygen *via* a nose mask with a bite-bar and continuous intramuscular infusion of dexmedetomidine (0.03 mg/kg/h). The rat was fixed to a custom-made holder to minimize the head motion during the whole scanning process. After the anatomical localization scans were acquired, the isoflurane concentration was decreased to 0.20–0.25% to keep the respiration rate at the range of 60–85/min throughout the scanning process. A small animal monitoring system (Model 1025, Small Animal Instruments Inc., New York, NY, USA), including a rectal temperature probe, respiration pneumonic sensor, and fiber optic oximetry sensor or cardiogram electrodes, was adopted for real-time monitoring. The core body temperature was maintained at 37°C *via* a warm water circulation system. If the respiration rate increased to 90/min, the concentration of isoflurane was increased to 0.5%.

Anatomical images (T2WI) were acquired with fast-spin–echo sequence using TurboRARE with the following parameters: repetition time (TR) 5000.0 ms, echo time (TE) 36.0 ms, echo spacing 12 ms, echo-train length 8, field of view 3.50 × 3.50 cm, matrix size 256 × 256, and 28 slices with a thickness of 1.0 mm. For blood-oxygen-level-dependent (BOLD) images, EPI-SE-FOVsat sequence was used with the following parameters: matrix size 64 × 64, flip angle = 90°, resolution = 0.55 × 0.55 mm, 28 slices with a thickness of 1.0 mm, slice gap = 0, TR = 2000.0 ms, TE = 18.0 ms, volume = 180.

### Data Processing and Analysis

The data were preprocessed using spmratIHEP software based on the statistical parametric mapping (SPM12) software and rs-fMRI Data Analysis Toolkit (REST) software, and statistically analyzed by spmratIHEP based on SPM12. The data were carefully examined for completeness and truncation artifacts. The FC values were analyzed and compared between the V group, SS-31 group, LPS group, and LPS + SS-31 group. All the functional images post-processing was performed by a single experienced observer, unaware to whom the scans belonged. The preprocessing and data analysis were performed using spmratIHEP (Nie et al., 2013, 2014) based on SPM12 (Welcome Department of Imaging Science; http://www.fil.ion.ucl.ac.uk/spm) and REST software (http://restfmri.net/Forum/Index.php?~q=rest).

The voxel size of the functional datasets of all individuals was first multiplied by a factor of 5 to better approximate human dimensions, and then preprocessed using the following main steps: (1) slice timing: The differences of slice acquisition times of each individual were corrected using slice timing. (2) Realign: The temporal processed volumes of each subject were realigned to the first volume to remove any head motion, and a mean image was created over the 180 realigned volumes. All participants had <1 mm of translation in the *x, y*, or *z* axis and a 1° of rotation in each axis. (3) Spatial normalization: The realigned volumes were spatially standardized into the Paxino and Watson space (Liang et al., 2017) by normalizing with the EPI template *via* their corresponding mean image. Subsequently, all the normalized images were resliced by 1.0 × 1.5 × 1.0 mm voxels (after zooming). (4) Smooth: The normalized functional series were smoothed with a Gaussian kernel of 2 mm^3^ full width at half-maximum (FWHM). (5) Removal of the linear trend: The smoothed images had any systematic drift or trend removed using a linear model. (6) Filtering: The band pass was filtered at 0.01–0.08 Hz as the physiological spontaneous BOLD fluctuation mainly focused on this band, and to remove the very low frequency drift and high frequency noise. The pre-processed images were analyzed within spmratIHEP in SPM12 based on the framework of the general linear model.

The FC analysis was performed by REST software (http://restfmri.net/Forum/index.php? q=rest) by placing seed regions at the left and right hippocampus (L- and R-hip). Pearson's correlation was computed between each voxels of the L- and R-hip, and the other intercranial voxels to obtain FC maps for each rat. To identify differences in the FC maps between groups V, S, L, and L + S, a one-way ANOVA and *post-hoc* two-sample *t-*test were performed. Regions with significant FC changes between each two groups were yielded based on voxel-level height threshold of *p* < 0.001 and a cluster-extent threshold of 20 voxels.

### Morris Water Maze

One day after treatment, learning and spatial working memory were assessed by the Morris water maze (MWM) with the same group of animals subjected to rs-fMRI scanning (*n* = 8–10 per group). Detailed description of the training protocols can be found in our previous study (Kan et al., 2016). Briefly, the MWM test was divided into two parts: spatial acquisition trials (day 1–5) and probe trial (day 7). On day 6, the rats were allowed for a rest. The water maze is a black painted circular tank with 150 cm in diameter and 60 cm in height, placing at the center of a quiet room. On the wall of the tank, there were four different visual clues, dividing it into four quadrants. A black curtain was held in place by a rectangular scaffold around the water maze to minimize harsh lights entering the room. A video camera mounted to the ceiling above the water tank was used to track the rat swimming speed, latency to find the platform, and target quadrant crossovers with an animal activity tracking system (Ethovision, Noldus Information Technologies, Wageningen, The Netherlands). For each training session, the tank was filled with clean water for about 35 cm in depth (23 ± 2°C) with a hidden circle platform 1.5 cm below the water surface at the third quadrant. On day 1–5, the rat was gently placed in one quadrant facing the wall of the MWM before each spatial acquisition trials. Then, the rat was allowed to swim freely for up to 60 s in the water maze to locate the platform. If the rat succeeded, it was allowed to stay for 5 s on the platform. Otherwise, the rat was physically placed on the platform for 20 s. Four spatial acquisition trials were performed on each rat per day and continued for 5 consecutive days. A 3–5 min interval was allowed between each trial. On day 7, a probe trial was conducted in which the platform was removed and the rat was placed at the quadrant opposite to the platform and allowed to swim for 30 s. The times the rat crossover the platform location and the percentage of time spent in the platform quadrant were recorded. From day 31–35, spatial acquisition trials were repeated followed by a probe trial on day 37. The time line for the MWM testing is shown in Figure 1B.

### Statistical Analysis

All data were analyzed using IBM SPSS 25.0 (IBM, Chicago, IL, USA). Data are presented as mean ± SEM. Results in the MWM spatial acquisition trials were analyzed by two-way ANOVA for repeated measurements. Results in the MWM probe trials were analyzed by one-way ANOVA. The above data were further analyzed by the Bonferroni *post-hoc* analysis. Other data were analyzed by two-way ANOVA followed by the Bonferroni *post-hoc* analysis.

For rs-fMRI data, the analyzing method is mentioned in section Data Processing and Analysis. To further determine the relationship between FC and performances in the behavior test, Pearson's correlation was performed. Prior to the correlation analysis, *t-value*s with statistical significance were transformed to Z-scores using the Fisher transformation.

## Results

### Lipopolysaccharide Exposure Impairs While Elamipretide (SS-31) Preserves Learning and Spatial Working Memory

The MWM was used to test the effects of LPS exposure and SS-31 treatment on learning and spatial working memory in both the short (<7 days) and long terms (>30 days). From day 1 to 5, all rats showed significantly improved escape latency during the 5 days training [*F*_(3,141)_ = 11.237 for treatments and *F*_(4,188)_ = 73.179 for training days, *p* < 0.001, repeated measures two-way ANOVA; Figure 1C]. Significant interactions between training days and treatments were also observed [*F*_(12,564)_ = 1.791, *p* = 0.046, repeated measures ANOVA]. The *post-hoc* analysis indicated that LPS caused a significant increased escape latency from day 2 (*p* < 0.001), while SS-31 showed a significant improved escape latency from day 4 compared with the LPS group (*p* < 0.001). For the probe trial on day 7, a significant difference in the time spent in the target quadrant (one-way ANOVA, *F* = 12.152, *p* < 0.001, Figure 1D) and platform crossovers (one-way ANOVA, *F* = 11.525, *p* < 0.001, Figure 1E) was observed. The *post-hoc* analysis revealed that the the LPS-treated rats spent significantly less time in the platform crossovers than vehicle, LPS + SS-31, and SS-31 treated rats (*p* < 0.001, *p* = 0.013, and *p* = 0.004, Figure 1E respectively). However, there was no difference in the time spent in target quadrant when comparing LPS exposure rats with either SS-31- (*p* = 0.159) or LPS + SS-31-treated rats (*p* = 0.063, Figure 1D).

For the second acquisition trials from day 31 to 35, there was no interaction between training days and treatments [*F*_(12,468)_ = 0.732, *p* = 0.720; Figure 1F]; however, there was a significant difference among training days [*F*_(4,156)_ = 6.790, *p* < 0.001]. The *post-hoc* analysis revealed that rats with the LPS exposure had a significantly different escape latency on day 35 compared with other groups (*p* = 0.002). For the probe trial on day 37, there was a significant difference in the time spent in the target quadrant (one-way ANOVA, *F* = 12.926, *p* < 0.001; Figure 1G) and platform crossovers (one-way ANOVA, *F* = 7.568, *p* < 0.001; Figure 1H). The *post-hoc* analysis revealed that the LPS-exposed rats performed fewer platform crossovers than rats treated with vehicle (*p* < 0.001), rats treated with SS-31 (*p* = 0.004), and rats treated with LPS + SS-31 (*p* = 0.017). In addition, the LPS-treated rats spent significantly less time in target quadrant compared with vehicle- (*p* < 0.001) and SS-31-treated (*p* < 0.001) rats.

### Oxidative Responses at the Systemic Level and Hippocampus After LPS Exposure and SS-31 Treatment

**Figure 2:**
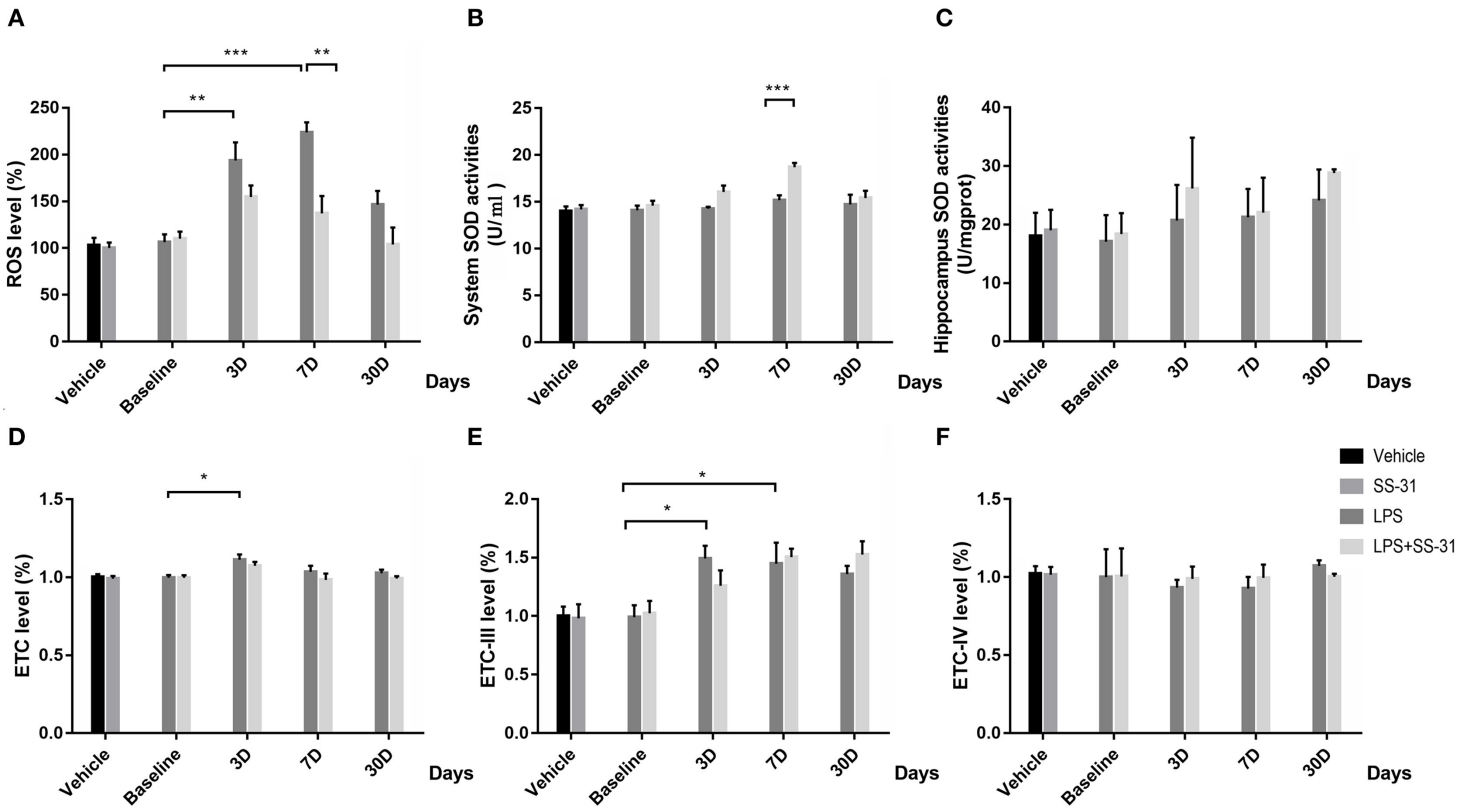
Effects of lipopolysaccharide (LPS) exposure and elamipretide (SS-31) treatment on oxidative stress. **(A)** LPS exposure caused a significant mitochondrial ROS (mtROS) increase while rats with SS-31 treatment have lower level. **(B,C)** LPS exposure and SS-31 treatment caused superoxide dismutase (SOD) changes in the hippocampus. **(D–F)** LPS exposure and SS-31 treatment caused electron transport chain (ETC) complex I/III/IV changes in the hippocampus. Data are presented as mean ± SEMs (*n* = 5). Comparisons were made by two-way ANOVA followed by the Bonferroni *post-hoc* analysis. *p < 0.05, **p < 0.01, ***p < 0.001 vs. LPS (L) group.

To determine the effect of SS-31 treatment in systemic level and aged rat hippocampus, oxidative stress was measured. For the hippocampus ROS, no interactive effect was observed [*F*_(3, 32)_ = 0.111, *p* = 0.111]; however, significant effects were seen in treatments and training days separately [*F*_(1, 32)_ = 10.775, *p* = 0.002 for treatment and *F*_(3, 32)_ = 8.167, *p* < 0.001, for days respectively; two-way ANOVA, Figure 2A]. Furthermore, the Bonferroni *post-hoc* analysis revealed that the hippocampus ROS started to increase 3 days after the LPS exposure (*p* = 0.009) and reached a peak level on day 7 (*p* < 0.001). On day 30, the ROS fell back to the baseline level (*p* = 0.705). Meanwhile, the hippocampus ROS was significantly higher as compared with rats receiving SS-31 treatment on day 7 (*p* = 0.002). For rats in L + S group, no significant change in the ROS level has been observed after the LPS exposure. For the SOD level, no interactive effect was observed [*F*_(3, 32)_ = 2.392, *p* = 0.087]; however, significant effects were seen in treatment and training days separately [*F*_(1, 32)_ = 13.212, *p* = 0.001 for treatment and *F*_(3, 32)_ = 6.040, *p* = 0.002, respectively; two-way ANOVA, Figure 2B]. No difference has been observed in the hippocampus SOD activities [*F*_(3, 32)_ = 0.523, *p* = 0.811, respectively; two-way ANOVA, Figure 2C]. Furthermore, a significant higher SOD level in the system level was observed in day 7 as compared with rats without SS-31 treatment (*p* < 0.001, the Bonferroni *post-hoc* analysis, Figure 2B).

For the ETC-I level, no interactive effect was observed [*F*_(3, 27)_ = 0.444, *p* = 0.723, respectively; two-way ANOVA, Figure 2D], while a significant effect was found in days [*F*_(3, 27)_ = 0.004, *p* = 0.723]. A significant rise of ETC-I level was observed on day 3 (*p* = 0.027, the Bonferroni *post-hoc* analysis), whereas no difference was observed on other time points. For rats with SS-31 treatment, no significant change in ETC-I was observed. For the ETC-III level, no interactive effect was observed [*F*_(3, 32)_ = 1.099, *p* = 0.364, respectively; two-way ANOVA, Figure 2E], whereas, a significant effect was observed in days [*F*_(3, 32)_ = 7.075, *p* = 0.001]. The ETC-III started to increase on day 3 and reached a peak level on day 7 after the LPS exposure (*p* = 0.025 and *p* = 0.05, the Bonferroni *post-hoc* analysis, respectively). On day 30, no significant difference could be observed (*p* = 0.182). For rats with SS-31 treatment, no significant effect could be observed.

No interactive effect or separative effect was observed in ETC-IV in both groups of rats [*F*_(3, 32)_ = 0.180, *p* = 0.909 for interactive effect, *F*_(1, 32)_ = 0.042, *p* = 0.840 for treatment and *F*_(3, 32)_ = 0.240, *p* = 0.868 for days, respectively; two-way ANOVA, Figure 2F].

### Inflammatory Responses at the Systemic Level and Hippocampus After the LPS Exposure and SS-31 Treatment

To determine the effect of the LPS exposure and SS-31 treatment, proinflammatory cytokines in the serum and hippocampus and the mRNA expressions were measured.

**Figure 3:**
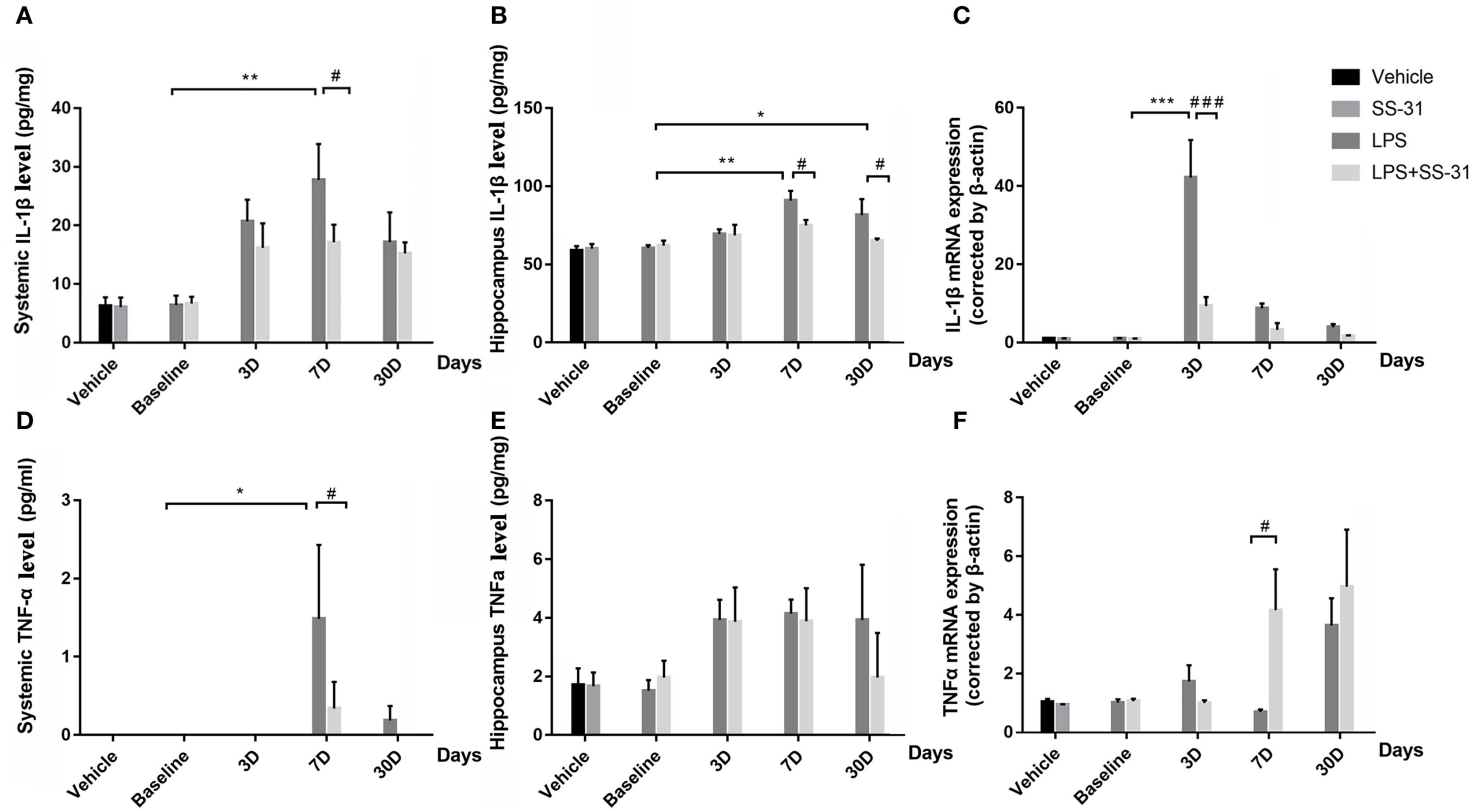
Effects of lipopolysaccharide (LPS) exposure and elamipretide (SS-31) treatment. ELISA showed differences in **(A,D)** the systemic and **(B,E)** the hippocampus IL-1β and TNFα expression. RT-PCR showed differences in mRNA expression for **(C)** IL-1β and **(F)** TNFα Data are presented as mean ± SEMs (*n* = 5). Comparisons were made by two-way ANOVA followed by the Bonferroni *post-hoc* analysis. ^#^*p* < 0.05, ^*##*^*p* < 0.01, ^*###*^*p* < 0.001 as indicated or vs. V; *p < 0.05, **p < 0.01, ***p < 0.001 vs. L group.

No interaction effects were seen in systemic levels in both IL-1β and TNFα [*F*_(3, 48)_ = 0.800, *p* = 0.500 and *F*_(3, 40)_ = 1.167, *p* = 0.334, respectively; two-way ANOVA]; while a significant effect was seen in days [*F*_(3, 48)_ = 6.514, *p* = 0.001 and *F*_(3, 40)_ = 1.167, *p* = 0.040, respectively; two-way ANOVA]. Furthermore, the Bonferroni *post-hoc* analysis revealed that the serum levels of IL-1β significantly increased and reached a peak level on day 7 (*p* = 0.001; Figure 3A) in rats with LPS. For rats with LPS + SS-31, no significant change in serum IL-1β levels was observed. When considering SS-31 treatment, a higher level of IL-1β was observed in the L group (*p* = 0.049, respectively). For TNFα, a significant increase was observed on day 7 (the highest increase, *p* = 0.034, respectively; Figure 3D). For rats with LPS + SS-31, no significant change in TNFα level was observed. On day 7, higher level of TNFα was observed in the L group (*p* = 0.030, respectively).

Further, proinflammatory cytokines in the hippocampus were analyzed for inflammatory responses in the central nervous system (CNS). No interactive effects were seen in both inflammatory cytokines [*F*_(3, 35)_ = 0.865, *p* = 0.469 for IL-1β and *F*_(3, 40)_ = 1.167, *p* = 0.334 for TNFα, respectively; two-way ANOVA], whereas significant effects in days were observed [*F*_(3, 35)_ = 5.260, *p* = 0.004 for IL-1β and *F*_(3, 40)_ = 3.033, *p* = 0.040 for TNFα, respectively; two-way ANOVA]. Furthermore, the Bonferroni *post-hoc* analysis revealed that the LPS exposure resulted in significantly increased levels of IL-1β on day 7 and 30 (*p* = 0.002 and *p* = 0.047, respectively, Figure 3B) as compared with the baseline level. For the effect of SS-31, significant lower levels of IL-1β were observed on day 7 and 30 after the LPS exposure (*p* = 0.041 and *p* = 0.034, respectively) when compared with the rats without SS-31 treatment. For TNFα, no significant change was observed though there was a tendency that the levels of TNFα seemed to be increased after the LPS exposure (Figure 3E).

When checking the mRNA expression in the aged rat hippocampus, a significant interactive effect in levels of IL-1β mRNA expression was observed [*F*_(3, 32)_ = 20.514, *p* < 0.001]. No interactive effect was seen in TNFα [*F*_(3, 32)_ = 1.651, *p* = 0.197, respectively; two-way ANOVA], whereas a significant effect was seen in days [*F*_(3, 32)_ = 4.251, *p* = 0.012]. To further determine the differences, the IL-1β mRNA expression reached a peak at day 3 after the LPS exposure (*p* < 0.001, the Bonferroni *post-hoc* analysis). When considering the effect of SS-31, a significant lower level of mRNA expression was observed on day 3 as compared with rats in L group (*p* < 0.001, Figure 3C). For TNFα, a significant difference of mRNA expression was observed on day 7 (*p* = 0.021, the Bonferroni *post-hoc* analysis, Figure 3F) when considering the SS-31 effect. For other time points, no significant differences in mRNA expression were observed for both groups of rats.

### Activation of Astrocytes in the Aged Rat Hippocampus After the LPS Exposure and SS-31 Treatment

To determine whether neuroinflammation in the aged rat hippocampus resulted in impaired behavior performances, the astrocyte activation in the inflammatory responses was measured.

**Figure 4:**
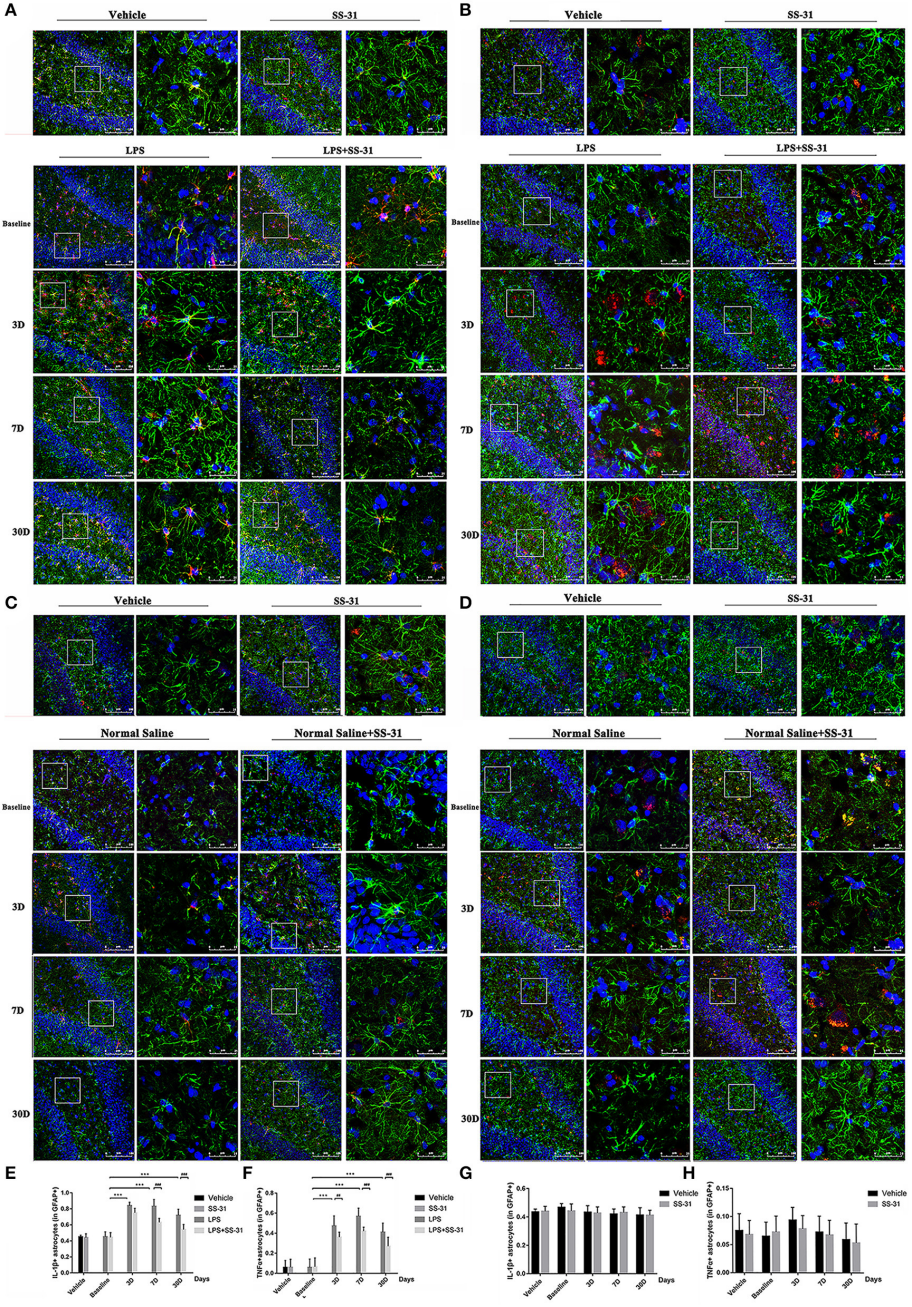
Astrocytes activation induced by lipopolysaccharide (LPS) exposure and elamipretide (SS-31) treatment. **(A,B)** LPS exposure induced a significant increase in IL-1β/TNFα secretion and astrocyte activation both in the LPS (L) and LPS + SS-31 (L + S) groups. SS-31 treatment improved the inflammatory response in the aged rat brain. **(C,D)** SS-31 treatment induced no significant inflammatory responses and astrocyte activation. **(E,F)** Double-stained immunofluorescence cell count showed that SS-31 treatment reduced the inflammatory response from day 3 to 30, whereas LPS caused prolonged IL-1β/TNFα secretion and astrocyte activation. **(G,H)** SS-31 treatment induced no significant change in IL-1β/TNFα positive astrocytes. Data are presented as mean ± SEMs (*n* = 5). Comparisons were made by two-way ANOVA followed by the Bonferroni *post-hoc* analysis. ^#^*p* < 0.05, ^*##*^*p* < 0.01, ^*###*^*p* < 0.001 as indicated or vs. V; *p < 0.05, **p < 0.01, ***p < 0.001 vs. L group.

In the hippocampus, both levels of IL-1β and TNFα secreted by astrocyte activation were affected by the LPS exposure and SS-31 treatment [*F*_(3, 72)_ = 3.371, *p* = 0.023 for IL-1β and *F*_(3, 72)_ = 4.691, *p* = 0.005 for TNFα, two-way ANOVA, Figures 4A,B]. When considering LPS exposure, the Bonferroni *post-hoc* analysis revealed that levels of IL-1β positive astrocytes were significantly increased on day 3, 7, and 30 in L group (*p* < 0.001, *p* < 0.001, and *p* < 0.001, respectively) and day 3 and 7 in L + S group (*p* < 0.001 and *p* = 0.001, respectively) compared with baseline. When considering SS-31 treatment, reduced levels of IL-1β positive astrocytes were observed on day 7 and 30 as compared with L group (*p* < 0.001 and *p* < 0.001, respectively; Figure 4E). For TNFα positive astrocyte, a significantly increased level was observed on day 3, 7, and 30 in L group (*p* < 0.001, *p* < 0.001, and *p* < 0.001, respectively) and L + S group (*p* < 0.001, *p* < 0.001, and *p* < 0.001, respectively). When considering the treatment effect, lower levels of TNFα positive astrocytes were observed in L + S group compared with rats in L group (*p* = 0.003, *p* < 0.001, and *p* < 0.001, respectively; Figure 4F).

For the treatment of SS-31, no significant difference has been found in IL-1β/TNFα positive astrocytes [*F*_(3,56)_ = 0.187, *p* = 0.987 for IL-1β and *F*_(3,56)_ = 0.060, *p* = 0.981 for TNFα, respectively; two-way ANOVA, Figures 4C,D,G,H].

### Hippocampal Neuroinflammation Affects the Hippocampus-Related Brain Function in Aged Rat

To further understand the effects of inflammatory responses in the hippocampus on the brain function in other regions, we used rs-fMRI scanning with L- and R-hip as seed voxels.

**Figure 5:**
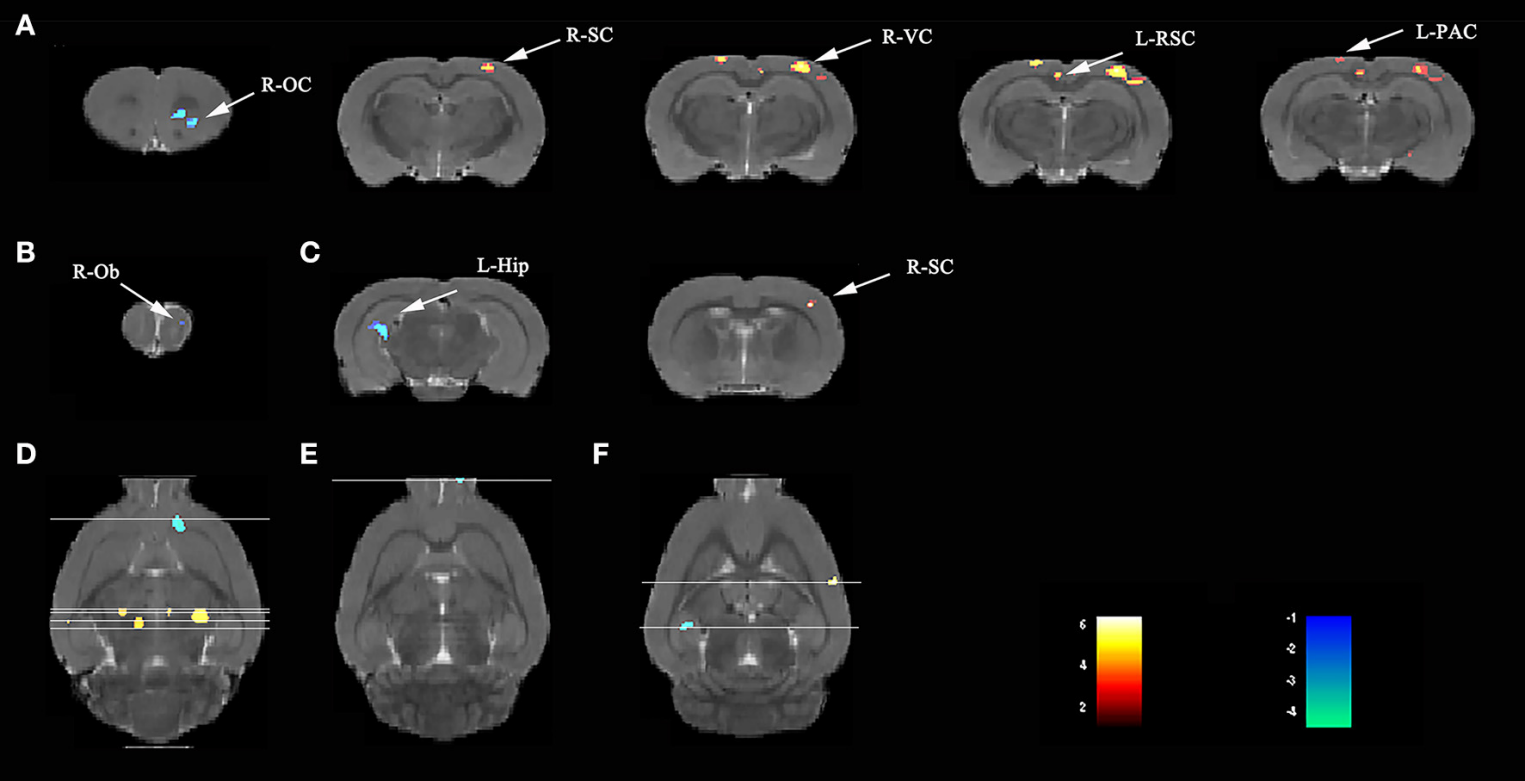
Lipopolysaccharide (LPS) exposure induced both short- and long-term functional connectivity (FC) changes in the aged rat brain. LPS exposure caused FC changes in **(A)** 3 days, **(B)** 7 days, and **(C)** 31 days. **(D–F)** Localization for different regions of interest (ROIs) on day 3, 7, and 31. Data were analyzed by one-way ANOVA followed by the *post-hoc* two-sample *t-*test. A voxel-level height threshold of *p* < 0.001 and a cluster-extent threshold of 20 were considered as statistically significant. Both the left and right hippocampus were used as seed voxels.

Compared with rats in the V group, those exposed to the LPS showed both decreased L-hip-seeded and R-hip-seeded FC on the right orbital cortex (R-OC), right olfactory bulb (R-Ob), and L-hip on day 3, 7, and 31, respectively (Figures 5A–C; Table 2). Using the same method, increased FC was observed on the right visual cortex (R-VC), right sensory cortex (R-SC), left retrosplenial cortex (L-RSC), and left parietal association cortex (L-PAC) on day 3 with the R-SC on day 31 (one-way ANOVA followed by the *post-hoc* two-sample *t-*test, *p* < 0.001, cluster 20; Figures 5A,C; Table 2). No FC increase was observed on day 7. Localizations in transverse plane are also presented (Figures 5D–F).

**Table 2:** LPS exposure caused significant functional connectivity (FC) changes in the aged rat brain in short and long term.

**Day**	**Cluster**	**Sub-regions**	**Cluster size**	***t*-value**	**Paxino's atlas**		
	**(Total)**				**x**	**y**	**z**
3	Cluster 1		80	−4.2917	2.4818	4.9159	3.0021
		Right-orbital cortex	80	−4.2917	2.4818	4.9159	3.0021
	Cluster 2		402	5.4930	3.2489	0.8785	−3.7179
		Right-visual cortex	87	5.1701	3.1171	1.0547	−4.1979
		Right-sensory cortex	110	5.4734	3.5018	0.7093	−3.2379
	Cluster 3		248	4.2736	−0.6197	1.1396	−4.1979
		Left-retrosplenial cortex	163	4.2736	−0.6197	1.1396	−4.1979
	Cluster 4		99	4.7769	−2.1034	−0.1988	−3.2379
		Left-parietal association cortex	69	4.7769	−2.1034	−0.1988	−3.2379
7	Cluster 1		164	−5.5104	1.4882	2.8678	7.3221
		Right-olfactory bulb	144	−5.5104	1.4882	2.8678	7.3221
31	Cluster 1		154	−5.1868	−4.4751	3.5214	−5.6379
		Left-hippocampus	154	−5.1868	−4.4751	3.5214	−5.6379
	Cluster 2		53	4.4788	5.6206	2.6984	−1.3179
		Right-sensory cortex	53	4.4788	5.6206	2.6984	−1.3179

*L, left; R, right. The regions of interest (ROIs) were drawn according to the rat anatomic atlas (Paxino and Watson, 4th edition).

*† The t-values were the maximum values of the two-sample t-tests for the ROIs with statistical significance (showing the greatest statistical significance within a cluster). A positive t-value means increased FC, while a negative t-value means the opposite. Comparison was performed for L and CON groups, with the V group as baseline. p <0.001 and a cluster size of 20 was considered as statistically significant*.

**Figure 6:**
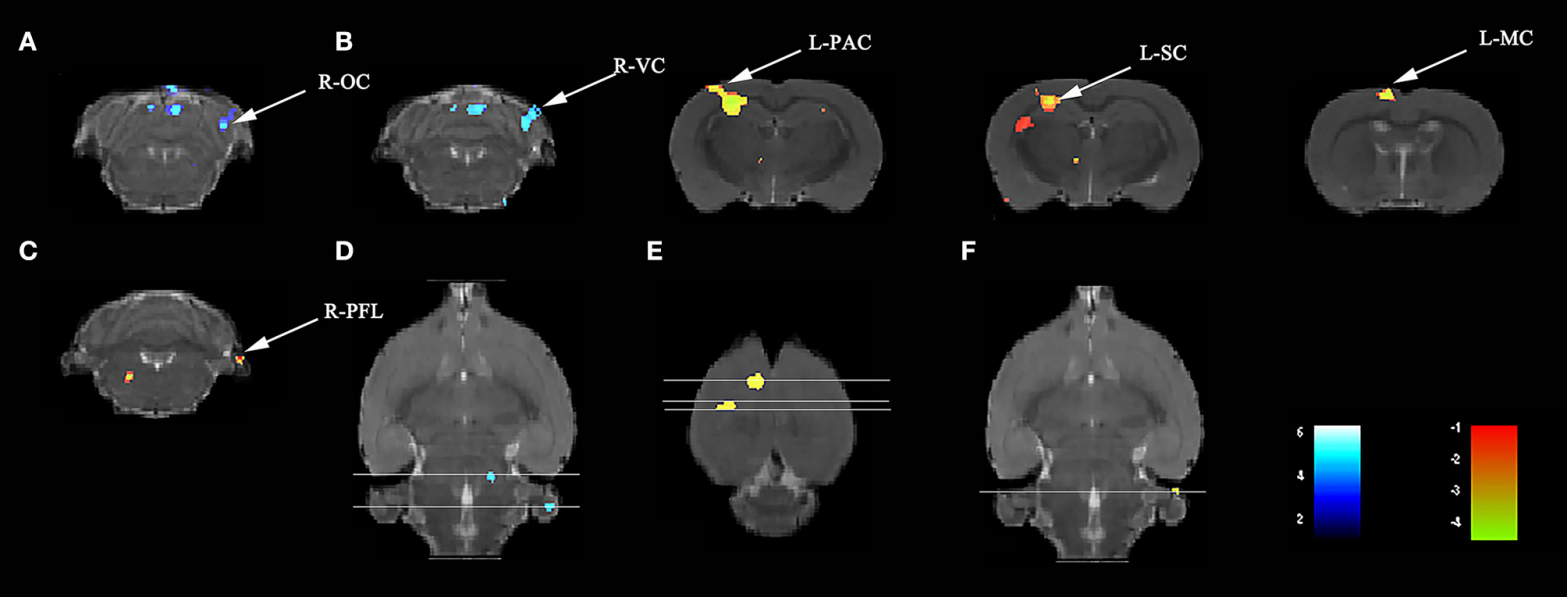
SS-31 treatment induced both short- and long-term functional connectivity (FC) changes in the aged rat brain. **(A)** SS-31 treatment caused FC changes in 3 days. SS-31 treatment improved FC connectivity in **(B)** 7 days and **(C)** 31 days. **(D–F)** Localization for different regions of interest (ROIs) on day 3, 7, and 31. Data were analyzed by one-way ANOVA followed by the *post-hoc* two-sample *t-*test. A voxel-level height threshold of *p* < 0.001 and a cluster-extent threshold of 20 were considered as statistically significant. Both the left and right hippocampus were used as seed voxels.

Compared with rats treated with LPS, those treated with LPS + SS-31 showed both increased L-hip-seeded and R-hip-seeded FC on the L-PAC, left sensory cortex (L-SC), and left motor cortex (L-MC) on day 7, and the right flocculonodular lobe (R-PFL) on day 31 (one-way ANOVA followed by the *post-hoc* two-sample *t-*test, *p* < 0.001, cluster 20; Figures 6B,C; Table 3). Using the same method, decreased FC was observed on the right olfactory cortex (R-OFC) and R-VC on day 3 (one-way ANOVA followed by the *post-hoc* two-sample *t-*test, *p* < 0.001, cluster 20; Figures 6A,C; Table 3). Localizations in transverse plane are also presented (Figures 6D–F).

**Table 3:** Elamipretide (SS-31) treatment improved functional connectivity (FC) connectivity in the aged rat brain.

**Day**	**Cluster**	**Sub-regions**	**Cluster size**	***t-*value**	**Paxino's atlas**		
	**(Total)**				**x**	**y**	**z**
3	Cluster 1		507	−5.9723	4.6668	3.1200	−9.9579
		Right-olfactory cortex	83	−5.3672	4.6602	3.2190	−9.4779
		Right-visual cortex	31	−4.4835	4.6536	2.8755	−8.9979
7	Cluster 1		709	5.9146	−3.1826	1.2896	−2.7579
		Left-parietal association cortex	61	5.3679	−3.3144	1.1826	−3.2379
		Left-sensory cortex	373	5.9146	−3.1826	1.2896	−2.7579
	Cluster 2		165	5.9085	−1.3406	0.2183	−0.8379
		Left-motor cortex	158	5.9085	−1.3406	0.2183	−0.8379
31	Cluster 1		32	4.781	5.7394	6.5273	−9.9579
		Right-flocculonodular lobe	21	4.781	5.7394	6.5273	−9.9579

*L, left; R, right. The ROIs were drawn according to the rat anatomic atlas (Paxino and Watson, 4th edition).

*†The t-values were the maximum values of the two-sample t-tests for the ROIs with statistical significance (showing the greatest statistical significance within a cluster). A positive t-value means increased FC, while a negative t-value means the opposite. Comparison was performed for L and L + S groups, with the L group as baseline. p <0.001 and a cluster size of 20 was considered as statistically significant*.

No significant FC change was found in V group and S group.

### Altered Brain Function Affected Learning and Spatial Working Memory

**Figure 7:**
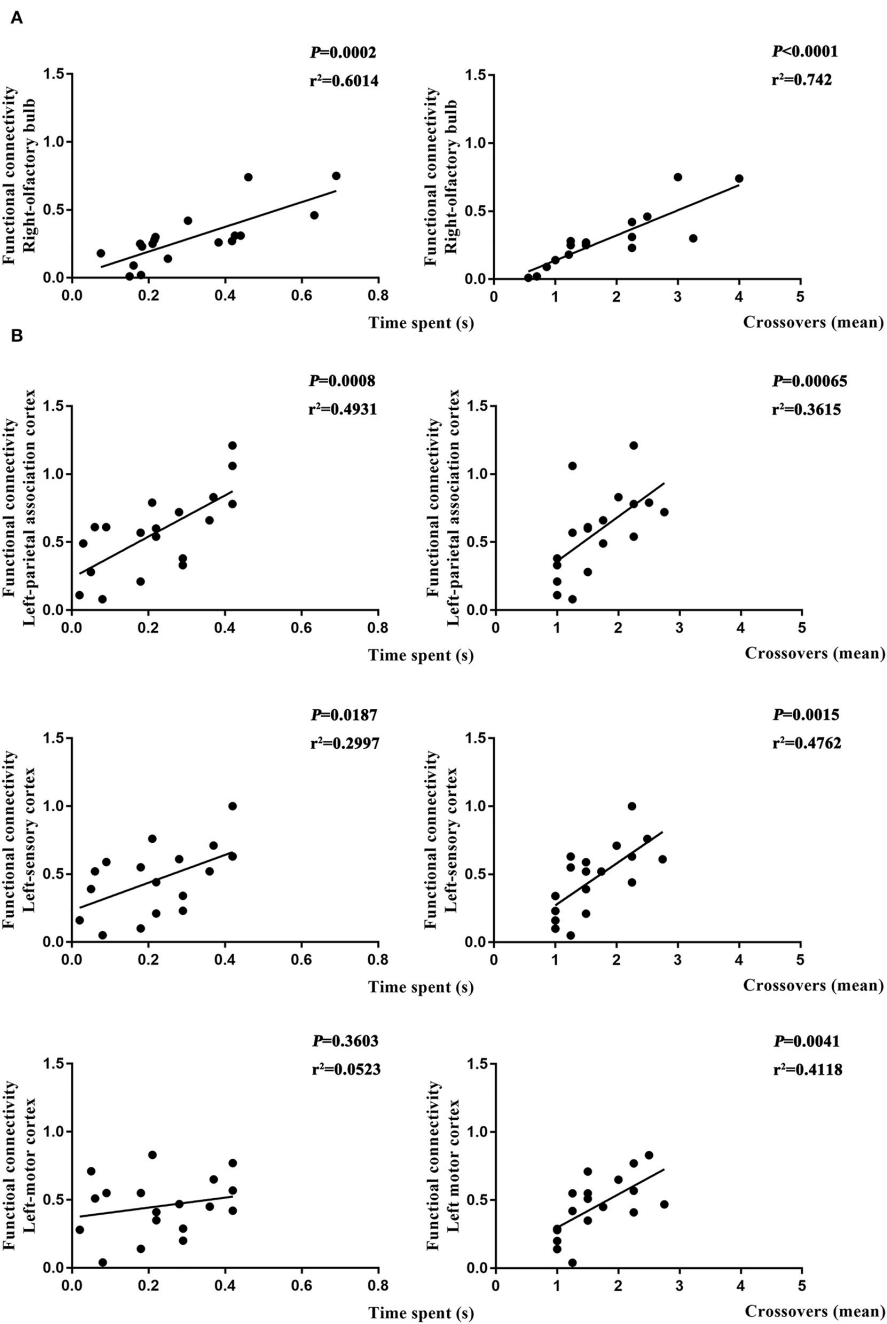
Relationship between functional connectivity (FC) and behavior performances on day 7. **(A)** Correlation analysis in L and NS groups. **(B)** Correlation analysis in L and L + S groups. Pearson's correlation was used with *p* < 0.05 for statistical significance and *r*^2^ for correlation.

To further determine if altered brain function in aged rat resulted in differences in learning and spatial working memory, the Pearson's correlation was used. On day 7, a significant correlation was observed in R-Ob in the L group (*p* = 0.0002, *r*^2^ = 0.6014 for time spent and *p* < 0.0001, *r*^2^ = 0.742 for crossovers, Figure 7A) as compared with rats in V group. Meanwhile, L-PAC, L-SC, and L-MC were also observed significant correlations with behavior performances (*p* = 0.0008 and *p* = 0.0187 with *r*^2^ = 0.4931 and *r*^2^ = 0.2997 for time spent; *p* = 0.00065, *p* = 0.015, and *p* = 0.0041 with *r*^2^ = 0.3615, *r*^2^ = 0.4762, and *r*^2^ = 0.4118 for crossovers, Figure 7B). However, no significant correlation was observed for L-MC on day 7 (*p* = 0.3603). On day 31, no significant correlations were observed for L-hip, R-SC, and R-PFL on latency (*p* = 0.0543, *p* = 0.1497, and *p* = 0.1997, Figure 8).

**Figure 8:**
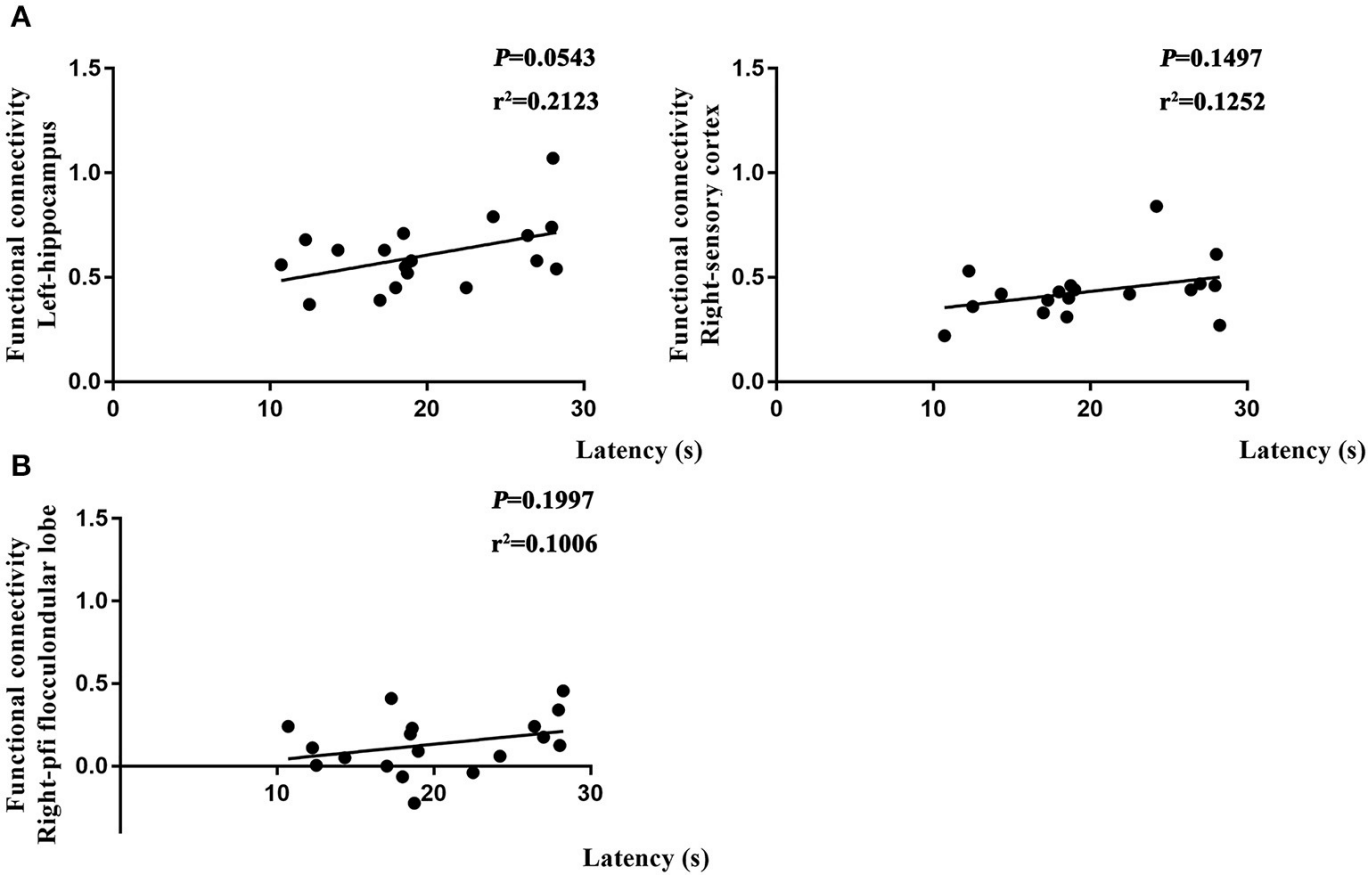
Relationship between functional connectivity (FC) and behavior performances on day 31. **(A)** Correlation analysis in L and NS groups. **(B)** Correlation analysis in L and L + S groups. Pearson's correlation was used with *p* < 0.05 for statistical significance and *r*^2^ for correlation.

## Discussion

Our result revealed that oxidative stress induced by a single dose of LPS injection may be able to cause long-term expression of astrocyte-derived inflammatory cytokines in the hippocampus, long-term FC change in the hippocampus, and other related regions, as well as impaired learning and spatial working memory. By removing excessive ROS triggered by the LPS exposure, elamipretide (SS-31) significantly reverse the above effects. Our study, therefore, demonstrates LPS-induced oxidative stress has long-term effects on the prolonged hippocampus neuroinflammation, cognitive function, and network connection between the hippocampus and other regions in aged rats.

The MWM is a classical approach to assess spatial learning and working memory. The spatial learning is assessed across repeated trials, and the reference memory is determined by preference for the platform area when the platform is absent (Vorhees and Williams, 2006). In our study, there is a significantly increased latency for rats in the L group as compared with rats in the other three groups, suggesting that the LPS exposure caused impaired learning ability in both short (<7 days) and long term (>30 days), and SS-31 treatment has a significant effect on improving the cognitive function. For reference memory, significantly decreased time spent in target quadrant showed cognitive dysfunction in both short (<7 days) and long term (>30 days). For rats with SS-31 treatment, significantly higher times for platform crossovers showed improved cognitive function. Although a higher time spent in target quadrant was observed, for rats in the S group, no significant difference was observed in time spent in target quadrant as compared with the rats with the LPS exposure. Considering the fact that the rats in the S group had already shown significantly higher times for platform crossovers and latency, with no significant difference in L-1β/TNFα positive astrocytes, the reason may be due to a rather strict correction in statistical analysis. Significant lower times for crossovers showed that the rats in the L group had significantly impaired reference memory.

Traditionally, astrocytes have been considered as a major contributor for neuron homeostasis, functional outcomes of local synaptic transmission and plasticity, and BBB constructing (Abbott et al., 2006; Santello et al., 2019). However, astrocytes have also recently been reported to play a pivotal role in the neuroinflammation and cognitive function (Habbas et al., 2015; Santello et al., 2019). We previously reported that a single systemic injection of LPS (2 mg/kg) activated NF-κB signaling pathway in astrocytes and induced long-term expression of IL-1β and TNFα mRNA in the aged rat hippocampus, which caused prolonged neuroinflammation (Fu et al., 2014; Kan et al., 2016). In addition, our study also reported that the LPS exposure caused decreased expression of post-synaptic density protein-95 (PSD-95) in neurons while blocking NF-κB signaling pathway in astrocytes improved the phenomenon (Kan et al., 2016). This demonstrated that the LPS exposure resulted in impaired synaptic plasticity, which further induced impaired learning and spatial working memory. In the present study, long-term neuroinflammation was inhibited by SS-31 treatment. Interestingly, SS-31 was specially designed to remove excessive ROS from mitochondria rather than inhibiting NF-κB signaling pathway (Wu et al., 2017; Zhao et al., 2019), suggesting that anti-oxidant treatment is also effective for inhibiting neuroinflammation in aged rat. Our result demonstrated that the SS-31 treatment inhibited LPS induced long-term astrocyte activation and enhanced expression of astrocyte-derived proinflammatory cytokines in the aged rat hippocampus, and it improved learning and spatial working memory. Although the A1 type astrocytes (neurotoxic and may contribute to neurodegenerative diseases) have been proved to be activated by microglia (Liddelow et al., 2017), recent studies also showed that astrocytes may trigger microglial activation (Jha et al., 2018). The present study showed that the LPS exposure induced astrocytes activation followed with long-term inflammatory responses in the hippocampus, suggesting that the astrocytes may play an important role in long-term inflammatory response in the aged brain. The previous studies revealed that mtROS generation may induce inflammasome activation in astrocytes (Alfonso-Loeches et al., 2014). The use of SS-31 treatment removed excessive ROS from the CNS, which helps to reduce the inflammatory response of the astrocytes, and further improved cognitive function in the long term. Overall, our findings suggested that anti-oxidant treatment may be a potential method for preventing POCD in elderly patients in clinical practice.

Mitochondrial reactive oxygen species induce the activation of NF-κB and the nucleotide-binding oligomerization domain (NOD)-like receptor family, pyrindomain containing 3 (NLRP3) inflammasome (Bauernfeind et al., 2009), which activate caspase 1 and result processing and secretion of IL-1β, IL-18, and TNF-α in the hippocampus of aged mice and cognitive impairments (Fu et al., 2020). For ROS, significant increase can be observed from day 3 to 7 with the treatment effect can only be seen on day 7. Our result demonstrated that the SS-31 treatment has a significant effect on the short term (within 7 days). For the SOD level, no significant effect can be observed in the hippocampus, though rats with SS-31 treatment seemed to have a higher level. The levels of ETC-I/III/IV have similar change, which is consistent with the previous findings that the ROS correlated with ETC-I/III/IV levels (Yan et al., 2020). The previous study on ROS level in the hippocampus also indicated a similar effect on ROS within 3 days after the LPS exposure (Zhao et al., 2019). Another study based on rat pups (7 days after birth) reported a similar effect on ROS production 6 h after isoflurane exposure (Wu et al., 2017). However, no results for long term were reported for both studies. Our result suggested that the inflammatory responses in the hippocampus last longer than oxidative stress. Early prevention of oxidative stress has a long-term effect on preventing inflammatory response in the aged rat hippocampus. However, the mechanism for oxidative induced long-term astrocytes activation still need further investigations.

It is an interesting phenomenon that although the compounds (LPS and SS-31) are no longer exist 30 days after administration, the inflammatory effect and treatment effect still exist in the aged rat hippocampus. Meanwhile, no long-term effect can be seen in solely SS-31 administration, but the effect of the LPS exposure is prevented. However, the mechanism should be further studied. The previous study reported a single dose of LPS injection (1 mg/kg, i.p.) is enough to make a long-term dysfunction of hypothalamic–pituitary–adrenal (HPA) axis in adult rats (2 months old), including a significantly increased TNFα in peripheral and corticosterone 4 weeks after the initial dose of LPS injection, which may likely to involve some kind of learning-like brain plasticity (Valles et al., 2002). In the present study, we used aged rats (20 months) with weaker BBB and less tolerance to peripheral stressors, and higher dose of LPS injection (2 mg/kg), the above which may cause a more severe inflammatory response. Another study based on single dose of LPS injection (2 mg/kg) reported that the neuronal function followed by systemic inflammation is NLRP3 inflammasome dependent (14 days after injection) in which the spine density of basal cell in the hippocampus CA1 region still remains significantly decreased even 3 months after the LPS exposure (Beyer et al., 2020). Meanwhile, a great number of evidence has shown that the NLRP3 inflammasome activation also contributes to aging process (Meyers and Zhu, 2020), which also supports our result that the inflammatory responses in the aged rat hippocampus may be long-lasting even the LPS and SS-31 no longer exist. Although we did not measure the level of NLRP3 inflammasome, we used the same doses and administration method of LPS. The long-term neuroinflammation may also be induced by the NLRP3 inflammasome. Based on the findings that the difference in oxidative stress only lasted for 7 days (ROS level and ETC-III level), there may conclude that early prevention of excessive oxidative stress may have a long-term effect in the inflammation prevention.

Although the rs-fMRI has been widely used to study cognitive function and neurodegenerative disease in human subjects, the functional basis of BOLD signal is still under debate. The previous studies in both resting or task fMRI revealed that the neural activity required for the execution of cognitive tasks corresponds to flow within a low-dimensional state space, which is further assumed to reflect the BOLD responses (Hutchison et al., 2013; Shine et al., 2019). Because studies found that the intrinsic connectivity networks under neural activity are likely shaped but not fully determined by structural connectivity (Damoiseaux and Greicius, 2009), other mechanisms may responsible for neural network activity. A clinical study based on post-mortem data revealed that the spatial pattern of neurotransmitter receptors density (e.g., dopaminergic D_1_ and cholinergic M_1_) and topology factors are closely related to neural activity at the network level (Shine et al., 2019), which suggests that the FC in rs-fMRI may also be related to neural activities from chemical transmitters besides axon and dendrites. The previous studies showed that the LPS exposure resulted in decreased amounts of PSD-95 (Kan et al., 2016), loss of neural transmitter receptors (Zhang et al., 2017), and swollen astrocytes (Fan et al., 2014), all of which may further impair synaptic plasticity (Santello et al., 2019). Therefore, as previously mentioned, the intrinsic connectivity networks under neural activity are likely shaped but not fully determined by structural connectivity (Hutchison et al., 2013), which indicates that changes in the brain function may occur in remote areas due to FC *via* neurotransmitters. In our study, rats exposed to LPS showed changes in FC not only in cognitive-related regions (e.g., L-RSC and L-hip) but also in regions distant from the hippocampus (e.g., R-Ob and R-OC); thus, neuroinflammation in the hippocampus may also affect remote regions *via* neurotransmitters rather than anatomical connectivity. This may also explain why the flocculonodular lobe is also engaged in the hippocampus-related brain function in aged rat despite located in cerebellum. Furthermore, our results suggest that anti-inflammatory treatment not only inhibits hippocampal neuroinflammation but also protects the brain function in other regions in the long term.

The default mode network (DMN) has been proved to be an important neural network, which activates during mind-wondering and becomes less active during task-acquisition (Raichle, 2015). The disruption of DMN is mostly seen in human neurocognitive disorders (Greicius et al., 2004; Qian et al., 2019). Meanwhile, the recent studies also found that the rat brain has DMN, and it has similar components as human beings (Lu et al., 2012). In the present study, rats with the LPS exposure showed changed FC in R-OC, L-RSC on day 3 and L-hip on day 31 with both sides of the hippocampus as seed regions. These regions are all major components of rat DMN. Imaging studies had already proven that different brain regions function as networks (Biswal et al., 1995; Wang et al., 2010), and a disruption of the brain connectivity suggested a long-term disruption for neural correlation between the hippocampus and target brain region. The RSC is situated at the crossroads between the hippocampus and neocortex (Wyss and Van Groen, 1991). Change in FC between Hip and RSC may result in information disruption in the aged rat brain. These may also explain why the rats still had impaired cognitive function 30 days after the LPS exposure. The prefrontal cortex (PFC) has been proved to be both significant in DMN and the hippocampus-prefrontal afferents that are critical for encoding memory (Raichle, 2015; Spellman et al., 2015). However, we did not observe a significant difference in FC between PFC and Hip. This indicated that the LPS exposure did not disrupt correlation between PFC and Hip. Considering that there are still significant differences in IL-1β secretion in the hippocampus, we hypothesize that this phenomenon may be resulted from the excessive IL-1β in the hippocampus. This is also consistent with the previous findings that IL-1β played a significant role in cognitive function (Huang and Sheng, 2010). However, in rats with SS-31 treatment, no significant change was observed in rat DMN though the improved MWM performance was observed. These results indicated that SS-31 may improve the brain FC by improving other regions, instead of DMN. It also suggested that DMN may not be the only network important in cognitive function. For the SS-31 treatment, FC change in the cerebellum was observed. Recent studies revealed that the cerebellum participates in both motor and cognitive aspects of navigation (Igloi et al., 2015; Wang et al., 2016). Our result is consistent with these findings and showed that improving FC between the hippocampus and cerebellum may also improve learning and spatial working memory.

Despite application of the latest medical practices, most of the neurodegenerative diseases remain difficult to cure and show irreversible development. Few studies have investigated whether inhibiting the hippocampus neuroinflammation or interrupting related signaling pathway improves the brain function in other regions. In one of our clinical studies, we reported improvements in cognitive test performance after anti-inflammatory treatment in elderly patients following spinal surgery (Zhang et al., 2018). However, rs-fMRI was not applied in this study so the function in other brain regions could not be fully assessed. In the present study, as consistent with improved behavior performances in the MWM, we observed increased FC from day 7 to 31 in the other brain regions using the bilateral hippocampus as seed voxels in rats treated with SS-31. Accordingly, we demonstrated that inhibiting inflammation in the hippocampus also improves the brain functions in other regions. Furthermore, IL-1β expression in the astrocytes on day 30 did not differ in rats with SS-31 treatment as compared with baseline data; however, a significant difference was observed on day 30 in L group. This indicates that SS-31 produces long-term protective effects against prolonged neuroinflammation in aged rat.

For the correlation analysis, our result demonstrated that the LPS exposure caused altered brain function is highly correlated with impaired behavior performances in the short term (<7 days). Interestingly, in the long term, altered brain function could be observed in both L and L + S groups as compared with rats in the NS group with no difference in behavior performances found. Moreover, a significant difference could still be found in probe trial (day 37) after the second MWM training session (day 31–35). Previous studies in human subjects reported abnormal PET or fMRI change at preclinical stage of cognitive diseases (Jack et al., 2010; Sperling et al., 2011), indicating that altered brain function may appear early than abnormal cognitive function as disease develops. For the present study, our result indicated that altered brain function may still exist in the long term after LPS exposure although the behavior may already seem to be back to normal. Moreover, our result suggested that these subjects (normal cognitive function with altered brain function) survived from neuroinflammation are still likely to have a worse cognitive function as compared with rats with SS-31 treatment or without LPS exposure.

There are several limitations for the present study. First, learning effect cannot be fully excluded. For groupwise comparisons at the same time point, the learning effect is minimized because each group underwent same training procedure; however, for the time-wise comparisons, the learning effect is a possibility. Studies demonstrated that the MWM training altered FC within DMN for up to 7 days in adult rat (Nasrallah et al., 2016). Whether 23 days (from day 7 to 31) is a sufficient test period for aged rat requires further investigations. Second, anesthesia could potentially influence FC patterns. Although a recent study demonstrated that combined isoflurane and dexmedetomidine anesthesia is an appropriate anesthesia method (Paasonen et al., 2018), data on the aged rat brain are still lacking because aging has also been reported to have influence on FC patterns (Ash et al., 2016). Third, the correlation analysis was done on time spent and crossovers in the short term (<7 days) and latency in the long term (>30 days). The reason for doing this is that the FC and behavior performance have to be at the same time so that the individual difference can be largely avoided.

In conclusion, our study demonstrated that the LPS-induced hippocampal neuroinflammation causes impaired brain function in other regions in aged rats, while inhibiting the inflammatory response in the hippocampus improves brain function in hippocampus and other related regions. The protective effect of inhibiting inflammatory responses coexisted with the improved behavior performance, fewer IL-1β- and TNFα-positive astrocytes in the hippocampus DG region, and lower IL-1β and TNFα secretion at the systemic level.

## Data Availability Statement

The raw data supporting the conclusions of this article will be made available by the authors, without undue reservation.

## Ethics Statement

The animal study was reviewed and approved by Ethical Committee of Capital Medical University.

## Author Contributions

YL, HF, and TW: author study design. YL, HF, FL, MK, and SY: experiments performance. YL, BN, and YW: data analysis. YL and HF: manuscript writing. TW, LF, and WX: manuscript revision. All authors contributed to the article and approved the submitted version.

## Conflict of Interest

The authors declare that the research was conducted in the absence of any commercial or financial relationships that could be construed as a potential conflict of interest.
