# Supplementary material for: Elamipretide (SS-31) Improves Functional Connectivity in Hippocampus and Other Related Regions Following Prolonged Neuroinflammation Induced by Lipopolysaccharide in Aged Rats
Source: Front Aging Neurosci. 2021 Mar 1;13:600484. doi: 10.3389/fnagi.2021.600484 (PMC7956963; doi:10.3389/fnagi.2021.600484)
Supplement: Supplementary file 1 [file Table_1.DOCX]

For microglia, no interactions for treatment and days were observed in both groups (*F*_(3,72)_ = 0.968, *p* = 0.424 for IL-1β and *F*_(3,72)_ = 2.187, *p* = 0.097 for TNFα, two-way ANOVA, Figure 1A and 1B). For LPS exposure, Bonferroni *post hoc* analysis revealed that levels of IL-1β positive microglia were significantly increased on day 3 in L group (*p* = 0.023) while no significant change were observed in L+S group. When considering the effect of SS-31, significant higher level of IL-1β positive microglia was observed on day 3 (*p* = 0.022). For TNFα, significant higher level of TNFα positive microglia was only observed on day 3 after LPS exposure in L group (*p* = 0.029, Figure 1E and 1F). No significant difference were observed in L+S group. When considering SS-31 treatment effect, no significant difference in TNFα positive microglia were observed.

For the treatment of SS-31, no significant difference has been found in IL-1β/ TNFα positive microglia (*F*_(3,56)_ = 1.460, *p* = 0.932 and *F*_(3,56)_ = 0.278, *p* = 0.841 for TNFα, respectively; two-way ANOVA, Figure 1C, 1D, 1G and 1H).


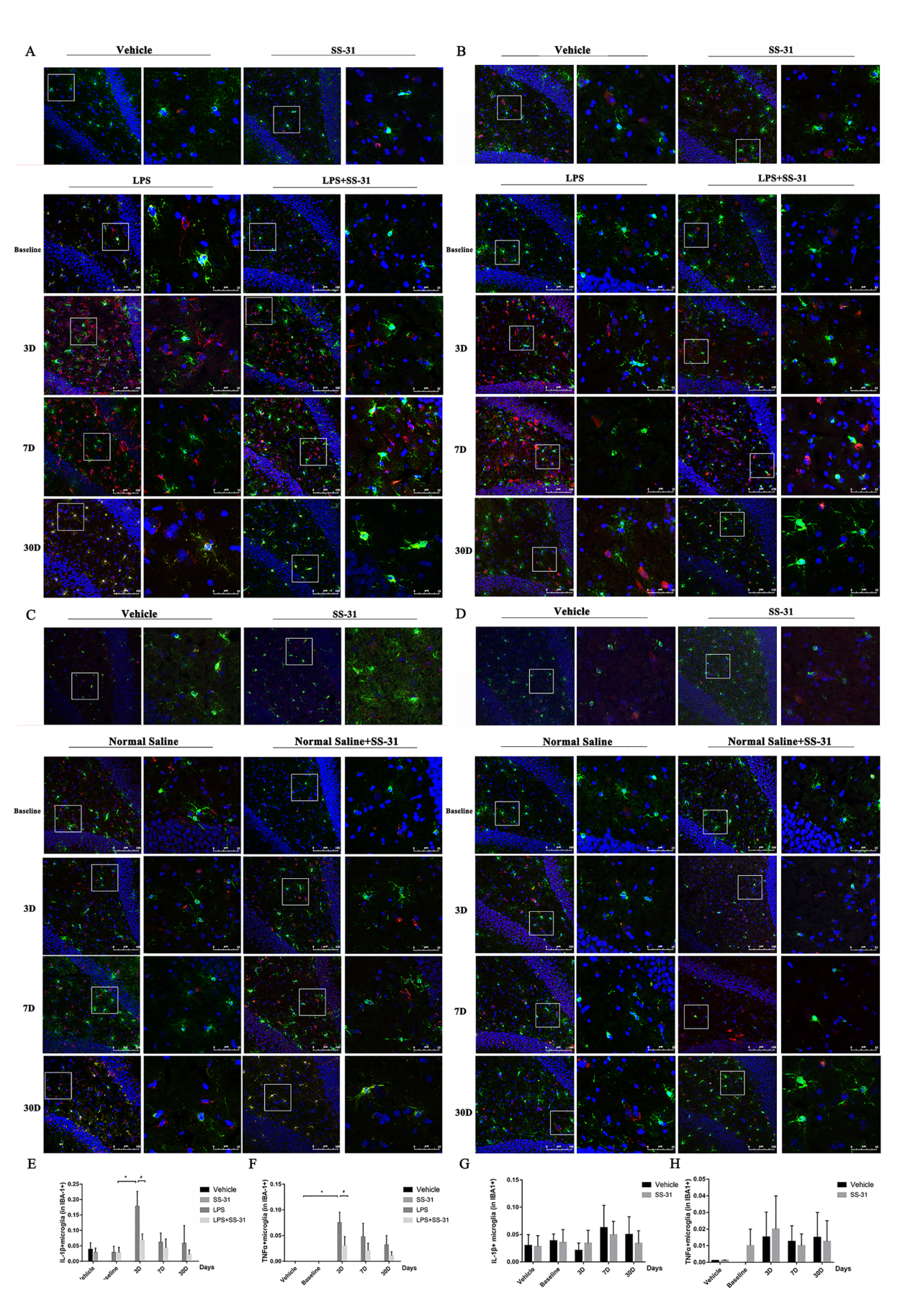


**Figure 1.** Microglia activation induced by lipopolysaccharide (LPS) exposure and elamipretide (SS-31) treatment. **(A, B)** LPS exposure induced a significant increase in IL-1β/TNFα secretion and microglia activation both in the LPS (L) and LPS+SS-31 (L+S) group. SS-31 treatment improved the inflammatory response in the aged rat brain. **(C,D)** SS-31 treatment induced no significant inflammatory responses and microglia activation. **(E,F)** Double-stained immunofluorescence cell count showed that SS-31 treatment reduced the inflammatory response, whereas LPS caused IL-1β/TNFα secretion and microglia activation only on day 3. **(G,H)** SS-31 treatment induced no significant change in IL-1β/TNFα positive microglia. Data are presented as mean±SD (n = 5). Comparisons were made by two-way ANOVA followed by Bonferroni *post hoc* analysis. ^#^*p* < 0.05, ^##^*p* < 0.01, ^###^*p* < 0.001 as indicated or *vs.* CON; ^*^*p* < 0.05, ^**^*p* < 0.01, ^***^*p* < 0.001 *vs.* L group

Because we had to do the mRNA analysis of these rats, we just re-do the system ELISA and found that there are no change in IL-1β after SS-31 treatment (*F*_(3,48)_ = 0.081, *p* = 0.970 for IL-1β, two-way ANOVA). Meanwhile, the TNFα level is under the detection line. I enclosed the systemic level of these two cytokines as attachments.


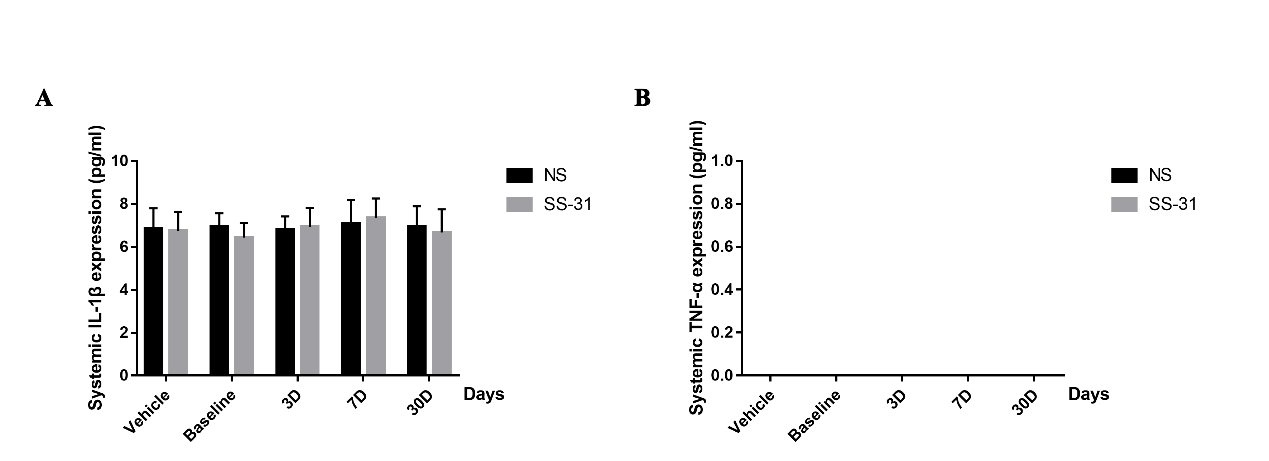


**Figure 2.** System level IL-1β and TNFα in Vehicle group and SS-31 treatment group. **(A)** SS-31 treatment didn’t cause a significant change in IL-1β. **(B)** TNFα is under detection line in both groups.


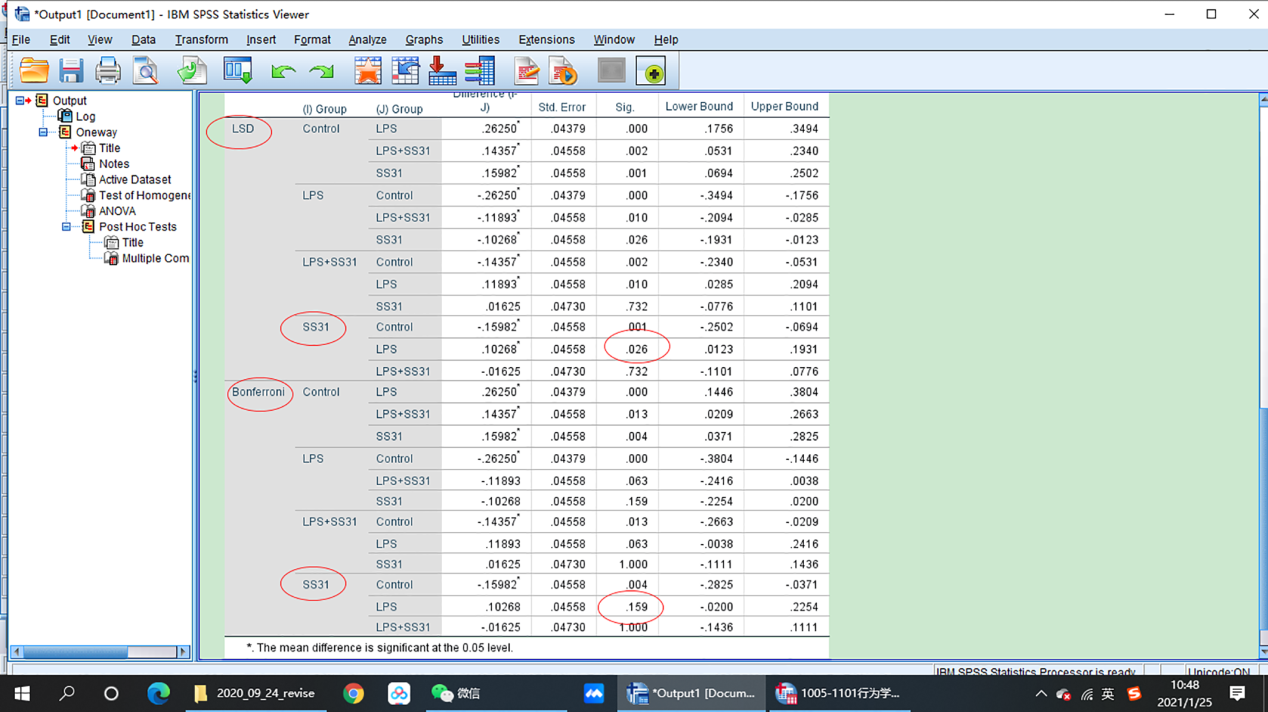


This is the process for collecting proteins from the hippocampus: The isolated hippocampus were homogenized in 100 mg tissue/ml RIPA lysis buffer (P0013C, Beyotime Institute of Biotechnology, Shanghai, China). Prior to use, the protease inhibitor (Solarbio, Beijing, China) were added into the RIPA lysis buffer. The resulting suspension were sonicated with an ultrasound homogenizer (Bandelin, OSTC) and further centrifuged at 12000 g for 4 min. The supernatants were collected and stored at -80℃ for further use.
